# A Tale of Two Diagnoses: Pulmonary Tuberculosis and Metastatic Lung Adenocarcinoma in a High-Risk Host

**DOI:** 10.7759/cureus.100775

**Published:** 2026-01-04

**Authors:** Anoohya Vangala, Shivani K Modi, Sankalp Acharya, Devarashetty Shreya, FNU Arty, Dheeraj K Pinninty, Sharon M Weiner, Shazia M Shah

**Affiliations:** 1 Internal Medicine, Monmouth Medical Center, Long Branch, USA; 2 Internal Medicine, Einstein Medical Center Philadelphia, Norristown, USA; 3 Internal Medicine, Jersey City Medical Center, Jersey City, USA; 4 Pulmonary and Critical Care, RWJBarnabas Health, Long Branch, USA; 5 Internal Medicine, Rutgers Health/Monmouth Medical Center, Long Branch, USA

**Keywords:** checkpoint inhibitors, lung adenocarcinoma, lung mass, smoking, tuberculosis

## Abstract

Tuberculosis (TB) and lung cancer are major global health burdens, often coexisting in high-risk populations such as smokers and immigrants from TB-endemic regions. We present a diagnostically complex case of a 57-year-old Filipino male with a chronic cough and a right upper lobe lung mass initially suspected to be TB. Imaging revealed spiculated nodules and widespread adenopathy, raising concern for malignancy. Although the initial biopsy was inconclusive, *Mycobacterium tuberculosis* was confirmed by polymerase chain reaction (PCR), and the patient was started on rifampin, isoniazid, pyrazinamide, and ethambutol (RIPE therapy). A repeat lymph node biopsy revealed metastatic lung adenocarcinoma with high PD-L1 expression (80%). He was treated sequentially with pembrolizumab followed by combination chemo-immunotherapy after disease progression. The patient tolerated treatment well, aside from nausea and an unrelated posterior cerebral artery infarct. This case underscores the diagnostic overlap and management challenges of coexisting TB and lung cancer and highlights the need for a multidisciplinary, staged approach in high-risk individuals.

## Introduction

Tuberculosis (TB) and lung cancer represent two of the most impactful global health challenges, each accounting for significant morbidity and mortality worldwide. TB remains a leading infectious cause of death, particularly in low- and middle-income countries, and continues to be highly prevalent in immigrant populations from endemic regions such as Southeast Asia [1]. Concurrently, lung cancer, particularly non-small cell lung cancer (NSCLC), is the leading cause of cancer-related deaths worldwide. The most common histologic subtype of NSCLC is adenocarcinoma [2].

The intersection of TB and lung cancer is not merely coincidental. Increasing evidence suggests a complex, bidirectional relationship in which chronic inflammation and immune evasion induced by TB may contribute to oncogenesis. At the same time, lung cancer and its treatments can exacerbate or unmask latent TB [3,4]. Moreover, the two diseases share several overlapping clinical manifestations, such as chronic cough, constitutional symptoms, and abnormal radiographic findings, including cavitary lesions or spiculated nodules. This often makes a timely and accurate diagnosis difficult [5]. 

In high-risk populations such as smokers, older adults, and immigrants from TB-endemic countries, the coexistence of these two pathologies must be carefully considered. Misdiagnosis or delayed recognition can lead to inappropriate treatment strategies, with potentially fatal consequences. Immunomodulatory treatments such as checkpoint inhibitors (e.g., Programmed Cell Death Protein 1 [PD-1] and Programmed Death-Ligand 1 [PD-L1] inhibitors) are commonly used in the treatment of lung cancer. These therapies have been associated with tuberculosis reactivation, further complicating disease management [6,7].

Herein, we present a diagnostically and therapeutically complex case of a 57-year-old Filipino male with active pulmonary TB and newly diagnosed metastatic lung adenocarcinoma. This case illustrates the overlapping presentations, diagnostic uncertainty, and treatment sequencing challenges that arise when managing dual pathologies.

## Case presentation

A 57-year-old Filipino male with a 30-pack-year smoking history (quit in 2015) was admitted to the hospital for evaluation of a right lung mass. He immigrated to the United States in 1995 and had a history of asthma, chronic obstructive pulmonary disease (COPD), and hypertension. His symptoms included a chronic cough with clear sputum for several months, but he denied hemoptysis, weight loss, or a family history of cancer. 

**Figure 1:**
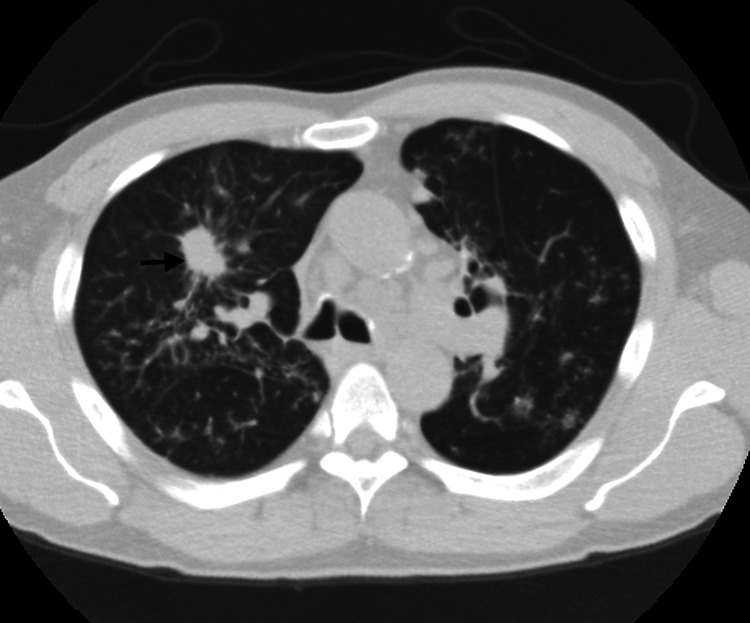
Axial CT chest showing right upper lobe nodule (black arrow)

**Figure 2:**
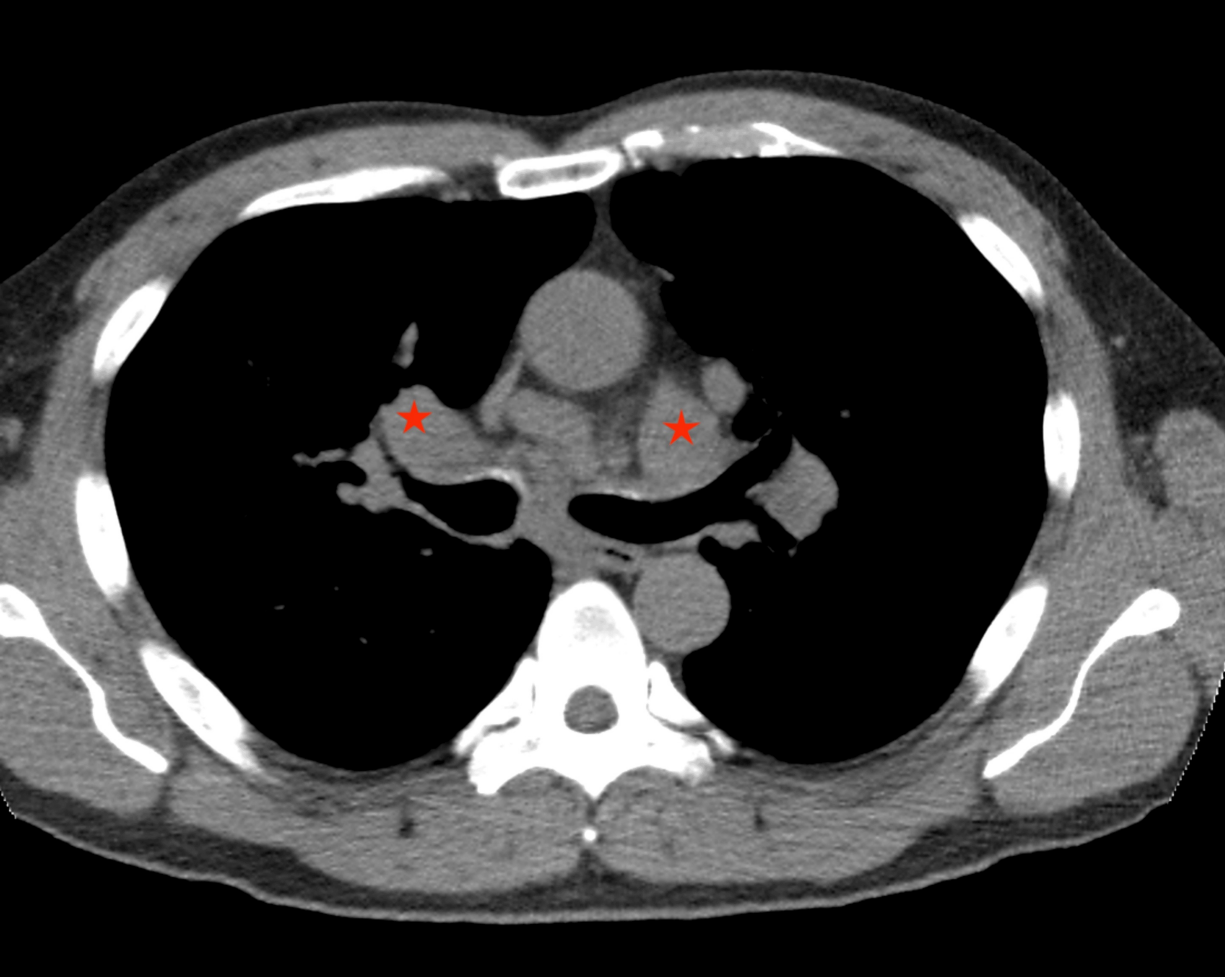
Axial CT chest showing bilateral hilar lymphadenopathy (red marks)

**Figure 3:**
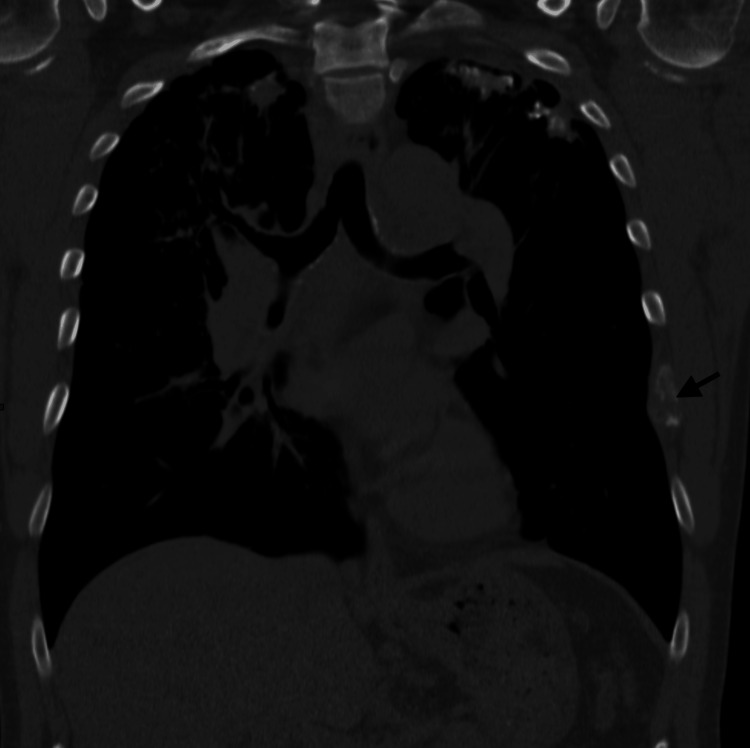
Coronal CT chest showing lytic lesion of the left lateral seventh rib (black arrow)

During the outpatient evaluation, low-dose computed tomography (CT) of the chest revealed a new spiculated 2.3 × 1.7 cm right upper lobe (RUL) nodule with newly enlarged mediastinal and hilar lymphadenopathy, suspicious of a neoplastic process (Figures 1, 2). A new lytic lesion involving the left lateral seventh rib with pathological fracture deformity was also seen (Figure 3).

**Figure 4:**
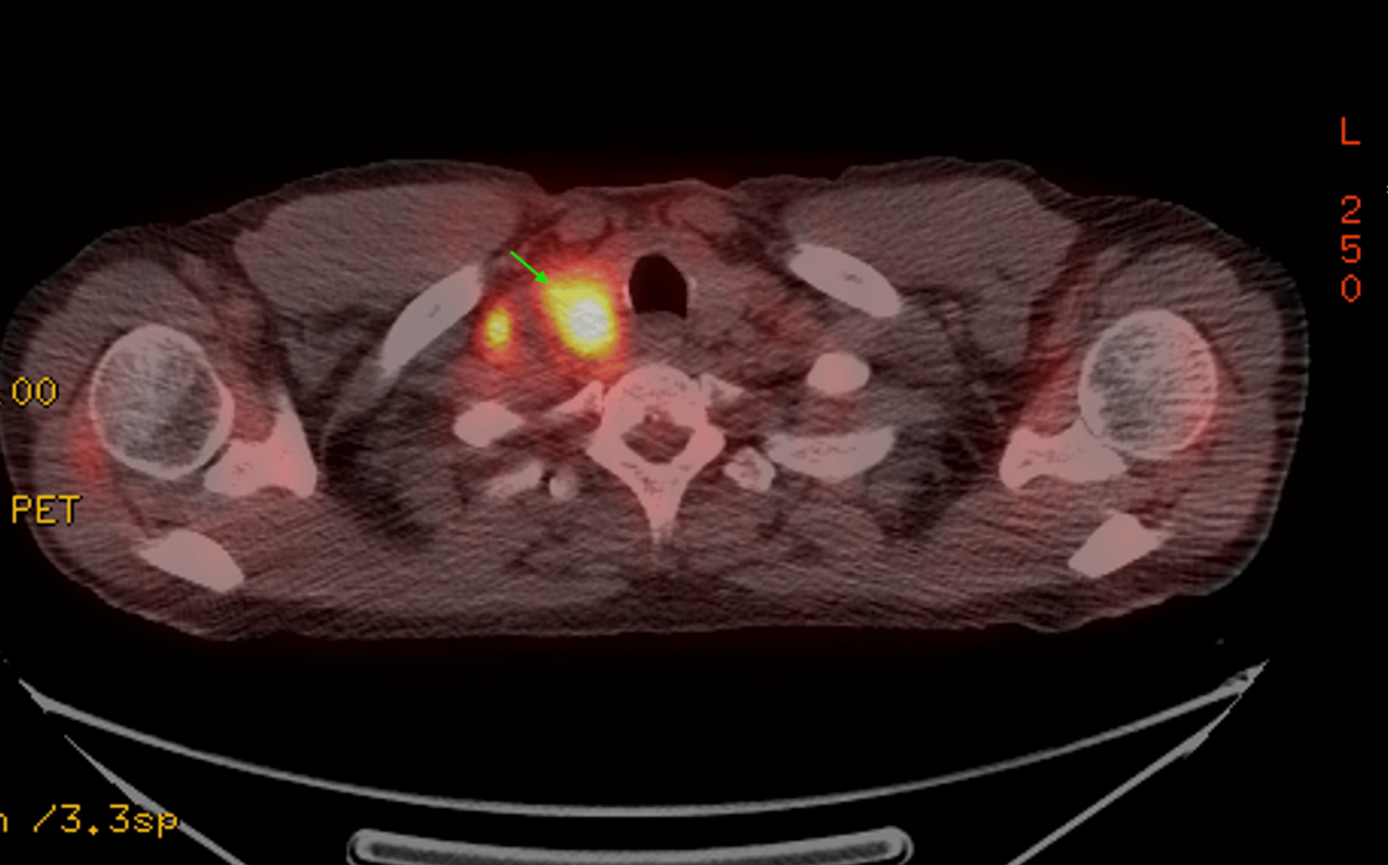
PET scan showing increased uptake in the RUL (green arrow)

**Figure 5:**
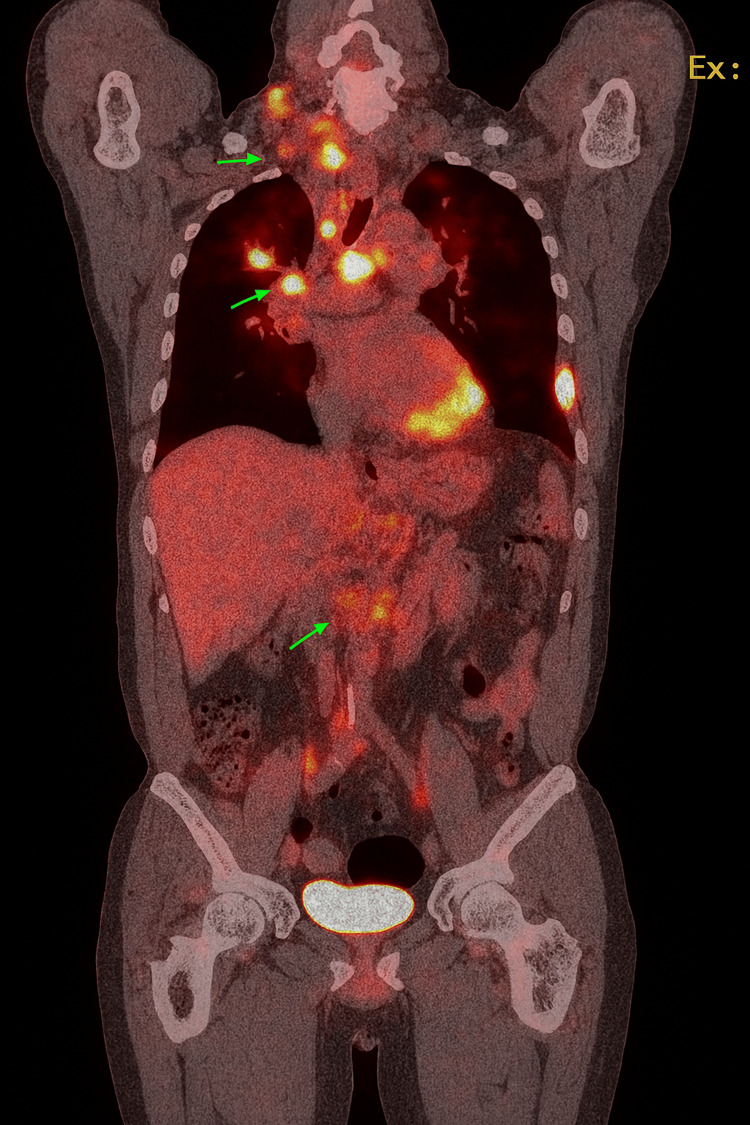
PET scan showing multiple areas of increased uptake suggestive of extensive malignant adenopathy in the neck, chest, and abdomen (green arrows)

Positron emission tomography (PET) demonstrated increased uptake in the RUL mass, highly suspicious of malignancy, along with extensive malignant adenopathy in the neck, chest, and abdomen (Figures 4, 5).

**Figure 6:**
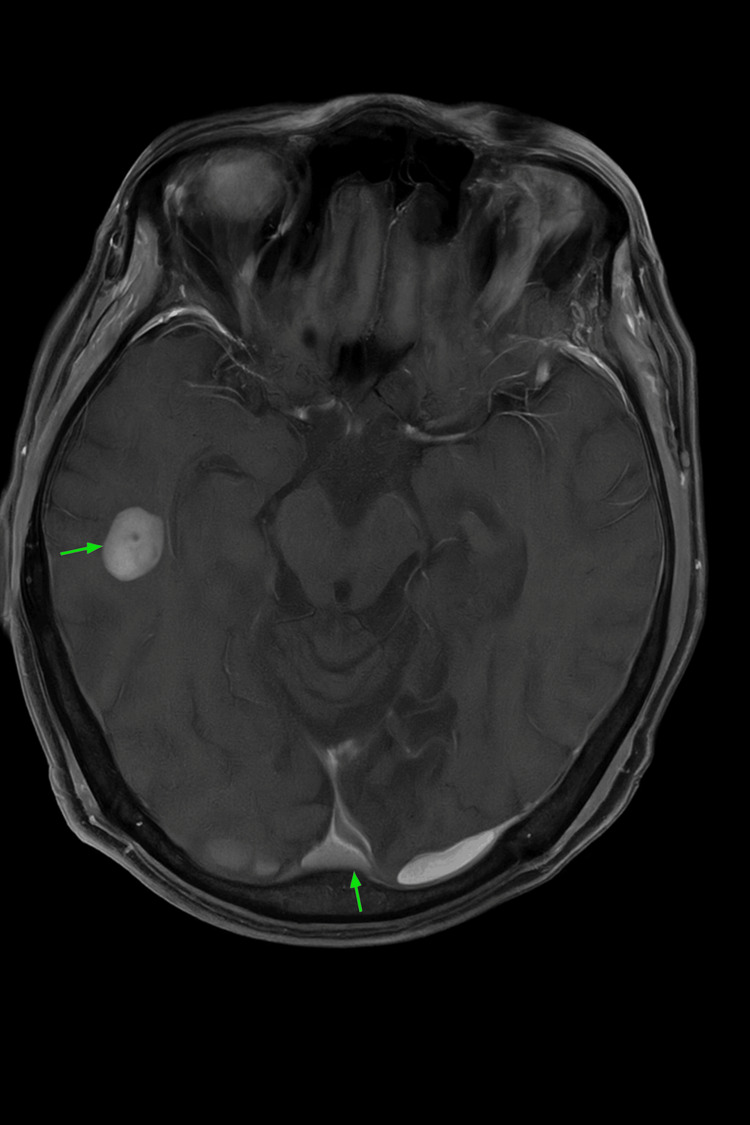
MRI of the brain showing enhancement in the bilateral supratentorial white matter and intracranial metastases without mass effect

Scattered uptake in the upper and mid-lung zones bilaterally was suggestive of either inflammatory changes or small metastases. Bony metastatic disease in the left lateral seventh rib was also noted. Magnetic resonance imaging (MRI) of the brain with contrast revealed scattered foci of enhancement in the bilateral supratentorial white matter, suspicious for intracranial metastases without mass effect (Figure 6).

A right cervical lymph node biopsy yielded fibroadipose tissue and was inconclusive. An acid-fast Bacillus (AFB) smear of sputum was negative. However, the patient’s QuantiFERON-TB Gold test resulted positive, raising suspicion for concurrent tuberculosis (TB). As a result, he was hospitalized with isolation precautions to expedite further evaluation of the lung mass.

Upon admission, vital signs were notable for a blood pressure of 163/88 mmHg and oxygen saturation of 96% on room air. Physical examination revealed cervical lymphadenopathy without auscultatory abnormalities. Laboratory results showed a white blood cell count of 7.6 × 10⁹/L.

A repeat AFB smear of sputum remained negative. However, sputum *Mycobacterium tuberculosis* polymerase chain reaction (MTB PCR) confirmed active TB infection. The patient was started on the anti-mycobacterial regimen: rifampin, isoniazid, pyrazinamide, and ethambutol (RIPE therapy).

Concurrent cancer workup with a repeat cervical lymph node biopsy confirmed metastatic lung adenocarcinoma (stage IV lung adenocarcinoma). The patient was evaluated by the State Health Department and discharged home for directly observed therapy (DOT) for a total of nine months. The decision regarding chemotherapy was deferred to the outpatient setting, pending molecular study results.

Four weeks later, molecular testing revealed high programmed death-ligand 1 (PD-L1) expression, measuring up to 80%. The patient was started on pembrolizumab (Keytruda) immunotherapy. After three cycles, a follow-up PET scan showed disease progression with new supraclavicular, axillary, mediastinal, and retroperitoneal lymphadenopathy, as well as new bone lesions. Treatment was subsequently escalated to a combination of carboplatin, pemetrexed, nivolumab (Opdivo), and ipilimumab (Yervoy).

During combined treatment with RIPE and chemotherapy, the patient generally tolerated therapy well, aside from expected chemotherapy-related nausea and vomiting. He was also hospitalized for an acute left posterior cerebral artery (PCA) territory infarct, although no direct correlation with treatment was identified.

## Discussion

This case highlights the complex interplay between lung cancer and active TB, two conditions that often coexist in regions with high TB burden and among individuals with significant risk factors such as smoking and immigration history. The patient, a 57-year-old Filipino male with a 30-pack-year smoking history, presented with a right lung mass initially suspected to be TB. This diagnostic challenge underscores the importance of maintaining a broad differential diagnosis in patients from high-risk populations, as TB and lung cancer share overlapping clinical and radiographic features [3].

The coexistence of TB and lung cancer is well documented, particularly in areas of high TB prevalence. Studies have shown that TB can mimic lung cancer radiographically, with findings such as spiculated nodules and lymphadenopathy, as seen in this case [5]. The positive QuantiFERON-TB Gold test and MTB PCR confirmation of active TB further complicated the diagnostic process, necessitating a comprehensive evaluation that included imaging, biopsies, and molecular testing. The eventual diagnosis of lung adenocarcinoma with high PD-L1 expression (80%) highlights the importance of advanced diagnostic tools in differentiating between these two conditions.

The management of concurrent TB and lung cancer presents unique challenges, particularly in balancing the treatment of an active infection with the need for oncologic therapy. In this case, the initiation of the RIPE regimen was critical to control the active TB infection and prevent progression during cancer treatment [8]. The decision to initiate immunotherapy with pembrolizumab, guided by high PD-L1 expression, reflects the growing role of precision oncology in complex cases. However, the use of immunotherapy in patients with active TB must be approached with caution, as it has been associated with immune-related adverse events and TB reactivation [6].

The coexistence of TB and lung cancer in this patient underscores the need for a multidisciplinary approach involving pulmonologists, oncologists, infectious disease specialists, and pharmacists. Close monitoring for treatment-related complications, such as hepatotoxicity from TB therapy and immune-related adverse events from immunotherapy, is essential. The patient’s acute left PCA territory infarct further illustrates the potential complications associated with managing coexisting serious illnesses and highlights the importance of vigilant monitoring. 

This case also provides insights into the epidemiology of TB and lung cancer. Immigrants from high TB-burden regions, such as the Philippines, are at increased risk of developing both conditions due to shared risk factors, including smoking, older age, and male sex [3,4]. The diagnostic delay in this case reflects broader systemic issues in healthcare access and delivery to immigrant populations, emphasizing the need for targeted interventions to improve early detection and treatment outcomes.

The therapeutic decision-making in this case raises important questions for future research. What are the optimal strategies for sequencing treatments in patients with concurrent TB and cancer? How can clinicians better predict and mitigate the risks of combination therapy in such cases? Emerging evidence suggests that sequential treatment approaches, as employed in this case, may offer better outcomes than concurrent therapy [7]. Additionally, the role of biomarkers such as PD-L1 in guiding immunotherapy decisions in patients with active infections warrants further investigation [9].

## Conclusions

This case highlights the diagnostic and therapeutic complexities of managing concurrent TB and lung cancer in a high-risk patient. Successful management requires a multidisciplinary approach, careful sequencing of treatments, and vigilant monitoring for complications. The insights gained from this case highlight the importance of comprehensive diagnostic evaluations, precision oncology, and targeted interventions to address the unique challenges faced by immigrant populations with complex medical histories.
